# 1-year data on patient-reported outcome is enough after surgery for degenerative cervical myelopathy: a cohort study from the Swedish Spine register

**DOI:** 10.2340/17453674.2024.42630

**Published:** 2025-01-09

**Authors:** Lovisa GERDHEM, Anna MACDOWALL, Paul GERDHEM

**Affiliations:** 1 Torsby Hospital, Torsby; 2 Department of Neurosurgery, Uppsala University Hospital, Uppsala; 3 Department of Orthopaedics and Hand Surgery, Uppsala University Hospital, Uppsala; 4 Department of Surgical Sciences, Uppsala University, Uppsala, Sweden

## Abstract

**Background and purpose:**

Degenerative cervical myelopathy (DCM) is the most common cause of spinal cord dysfunction in adults. Repeated follow-ups after surgery are resource consuming. The aim was to examine whether patient-reported outcome measures (PROMs) change after the first year. The purpose of this study was to investigate whether it is necessary to obtain follow-up data from patients more than 1 year after surgery for DCM.

**Methods:**

We included individuals treated surgically for DCM in the Swedish Spine registry (Swespine), with available preoperative, 1-, and 2-year PROMs, primarily the European Myelopathy Scale (EMS) and secondarily the Neck Disability Index (NDI), and the European Quality of life Visual Analogue Scale (EQ-VAS). A tertiary analysis included available 5-year data. Median, interquartile range (IQR), and Bland–Altman plots were used to compare PROM data at different follow-up time points.

**Results:**

642 individuals had baseline, 1-, and 2-year follow-up data, of whom 347 also had 5-year data. EMS was 14 (12–16) preoperative, 15 (12–17) at the 1-year follow-up, and 15 (12–17) at the 2-year follow-up. Corresponding data for NDI was 38 (24–50), 25 (12–42), and 26 (12–42) and for EQ-VAS 50 (30–60), 60 (42–77), and 60 (40–75). Similar findings were seen in individuals who also had 5-year data. Bland–Altman plots indicated good agreement between 1- and 2-year data, and between 1- and 5-year data and were without proportional bias.

**Conclusion:**

In individuals treated for DCM no clinically meaningful change in PROMs occurred after the 1-year follow-up.

Degenerative cervical myelopathy (DCM) is the most common cause of spinal cord dysfunction in adults [1]. It is often a progressive condition, with symptoms such as balance disorder, paresthesia and motor weakness in the extremities, and loss of fine motor skills. As it is a progressive disease, neurological function will deteriorate and lead to significant disability. The treatment of choice for DCM is surgical decompression, which aims to halt the disease progression and, to some extent, achieve recovery of function. Since randomized clinical trials (RCTs) are difficult to perform on this subject, observational studies using patient-reported outcome measures (PROMs) have become an alternative way of measuring outcomes of interventions.

Observational studies presenting data after 2 years of follow-up or longer are scarce, but it is possible that improvement occurs already in the first year after surgery [2-6].

Determining the timing of improvement after surgery would be beneficial to both clinicians, patients, and researchers. A shortened follow-up time would save resources that could instead be focused on improving response rates, which tend to decrease over time in cohort and registry-based studies [7].

The purpose of our study was to investigate whether there is further improvement in PROMs after the 1-year follow-up after surgery for DCM.

## Methods

### Study population and inclusion criteria

Data from the Swedish Spine registry (Swespine) was used for the study. The registry has a completeness of about 80% and a diagnostic accuracy of 97% [8]. Individuals 20 years of age or older with DCM due to cervical disc herniation or cervical spinal stenosis, with no previous cervical spine surgery, were included. Diagnosis was made by the treating surgeon and based on the presence of symptoms, clinical signs, and imaging findings indicating DCM. The surgeon noted preoperative neurological impairment. Included individuals had answered at least the European Myelopathy Scale (EMS) questionnaire at baseline, 1-year, and 2-year follow-up. Other available PROMs were the European Quality of life Visual Analogue Scale (EQ-VAS), Neck Disability Index (NDI), and Numeric Rating Scale (NRS) for neck and arm pain. In a tertiary analysis, we included individuals who had also responded to the EMS questionnaires at the 5-year follow-up. The study was reported according to the STROBE guidelines.

### Patient-reported outcome measures

The EMS is a self-reported questionnaire with 5 subscores, which are designed to evaluate different parts of the neural system that can be severely affected by DCM: upper motor neurons (gait function and control of bladder/bowel function), lower motor neurons (hand function), posterior roots (upper limb radicular deficits and paresthesia) and posterior columns (proprioceptive sensory loss, disturbed coordination, and ataxia). Minimum score is 5 points, maximum score is 18 points; the lower the score the more severe the deficits. Patients with 17–18 points are regarded as having normal function. The score is further classified in the following way: severe disease (5–8 points), moderate disease (9–12 points), and mild disease (13–16 points) [9,10]. The scale has been compared with the Patient-modified Japanese Orthopaedic Association scale (P-mJOA), which is a PROM that is more widely used internationally. It is based on the mJOA, which is a clinician-reported outcome measure. The PROMs EMS and P-mJOA have been found to be in agreement for milder disease, but EMS has lower sensitivity for severe disease compared with P-mJOA [11]. The minimal clinically important difference (MCID) has not been established for either of these 2 scales. MCID for mJOA has been found to be 1–2 points [12].

EQ-VAS is a visual analogue scale from 0 to 100 (best) for general quality of life [13,14]. MCID for EQ-VAS has not been defined for DCM, but was calculated to be 12 points for improvement and –7 points for deterioration in a study on lumbar disc herniation and spinal stenosis [15].

NDI is one of the most validated neck disease-specific outcome measures [16]. It is composed of 10 questions concerning the patient’s neck disability and how it is related to different aspects of everyday life. The higher the score, the more severe the disability. The total score is converted into a percentage from 0 to 100 (worst). MCID for NDI is 7.5 out of 50 points, and when converted to a percentage, 15 [17].

NRS for neck or arm pain ranges from 0, no pain, to 10 points, the worst imaginable pain. Previously, the Visual Analogue Scale (VAS) was used in Swespine. VAS data in the registry has been converted to NRS by dividing the VAS score by 10 with a stochastic approximation of decimals to the closest integer [18]. MCID for NRS has been defined as 2.5 points for neck and arm [19,20].

Patients report the maximum walking distance at baseline and at follow-ups (< 100 m, 100–500 m, 500 m–1 km, and > 1 km), here dichotomized to < 500 m and ≥ 500 m. Pain medication usage (yes/no), smoking (yes/no), duration of neck and arm pain was reported at baseline (no neck pain, < 3 months, 3–12 months, 1–2 years, or > 2 years). Data on sick leave (none, part-time, or full-time) was reported at baseline and at the follow-ups. At follow-ups, patients also reported whether they were satisfied, uncertain, or dissatisfied with the treatment.

### Statistics

A cohort with available EMS data at baseline, 1, and 2 years of follow-up was created. A subgroup of individuals who had also responded to the EMS questionnaires at the 5-year follow-up was also created. Missing data was handled through pairwise exclusion in the analyses. For continuous data, the number of responders is noted for each PROM in the table. Normality of continuous data was checked by visual inspection of histograms. Most of the data was not normally distributed, thus continuous data is presented as median with interquartile range (IQR). Categorical data is presented as number (%). To test for statistical significance, the Wilcoxon or Mann–Whitney tests were used for continuous variables, and the McNemar, sign test, or chi-square tests for categorical variables. The Spearman test was used to assess correlations, with the 95% confidence intervals (CI) based on the formula by Fieller, Hartley, and Pearson.

Agreement between EMS, EQ-VAS, and NDI at the follow-up time points 1 and 2 years, and 1 and 5 years were determined with Bland–Altman plots (means plotted against mean difference). Limits of agreement were defined as the range expected to include 95% of the differences between PROMs (+ 1.96 x standard deviation of difference), with the corresponding 95% CIs also depicted in the graphs [21]. We performed a mixed analysis of variance (ANOVA) to evaluate the effect of an interaction between the PROMs and follow-up time (at 1, 2, and 5 years), including the possible covariates, age and sex.

Analysis of non-responders was performed in the following manner. 1-year follow-up data for patients with data at baseline and 1 year were compared with those that had data at baseline, 1 year, and 2 years. 2-year follow-up data for patients with baseline, 1-, and 2-year data were compared with those that had data at baseline, 1, 2, and 5 years.

Statistical analyses were performed in IBM SPSS Statistics v28.0.1.1 (IBM Corp, Armonk, NY, USA) and in R Studio statistics software, version 2023.12.1. (R Foundation for Statistical Computing, Vienna, Austria)[22].

### Ethics, funding, use of AI, and disclosure

Swespine uses opt-out, meaning that patient consent is not needed to be part of the quality registry, but answering the questionnaires is voluntary. The Regional Ethical Review Board in Stockholm has authorized the study and the use of the data collection (numbers 2012/206/31-1 and 2018/2746-32). A de-identified dataset generated during the current study is available from the corresponding author on reasonable request. This study was supported by Värmland County in a clinical research appointment. The funding source had no role in the study design, analyses, or interpretation of data, in the manuscript writing, or in the decision to submit the paper for publication. Artificial intelligence was not used for the preparation of this manuscript. No benefits in any form have been received or will be received from a commercial party related directly or indirectly to the subject of this article. The authors have no conflicts of interest to report. Complete disclosure of interest forms according to ICMJE are available on the article page, doi: 10.2340/17453674.2024.42630

## Results

**Figure 1:**
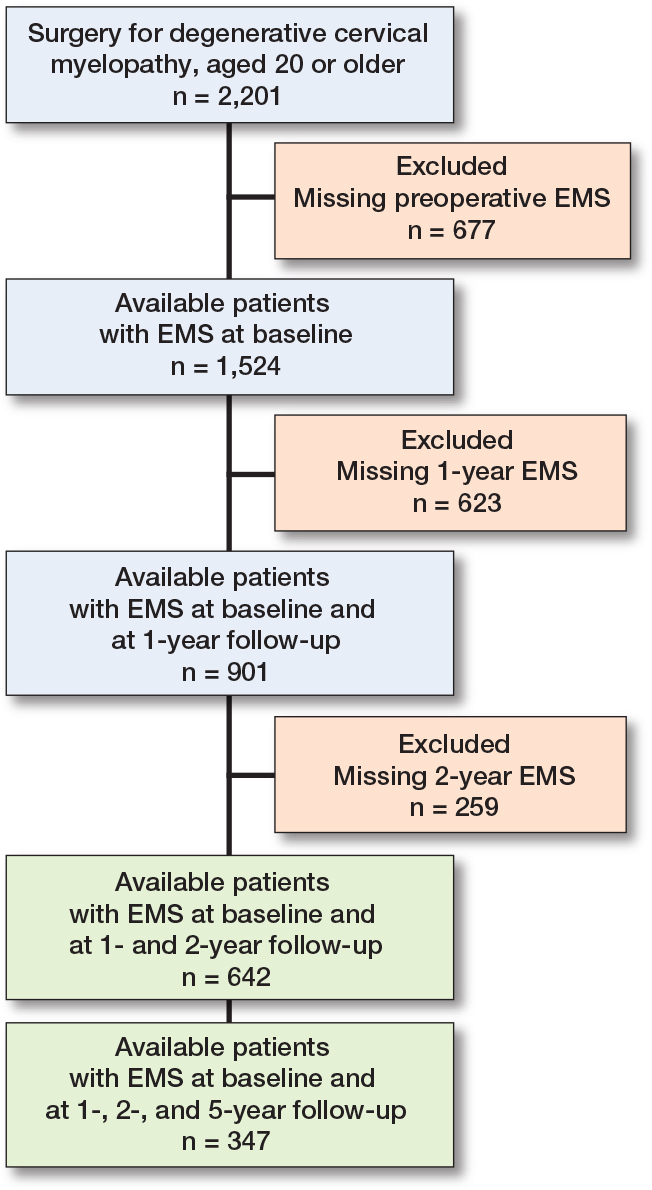
Patient flowchart.

A flowchart describing the inclusion process may be seen in Figure 1. 642 patients had baseline, 1-, and 2-year follow-up data on EMS, and 347 patients also had 5-year follow-up data. 634 of the patients had at least some preoperative motor function, while 8 did not. Baseline characteristics are presented in Table 1. Clinically relevant improvements in PROMs were seen between baseline and 1 year, but not after 1 year (Tables 2 and 3). There were some missing values for EQ-VAS, NDI, and NRS. The number of missing values is presented in the Tables for each PROM.

**Table 1:** Baseline characteristics for the 642 individuals with baseline, 1-, and 2-year follow-up data. Continuous data is presented as median (IQR), categorical data as n (%)

Age, median	65 (57–72)
Male sex, n (%)	383 (60)
Bodyweight (kg)	80 (69–90)
Body height (cm)	171 (166–179)
Body mass index	26.2 (24.1–29.3)
Smokers, n (%)	99 (15)
Duration of neck pain, n (%)	
No neck pain	140 (22)
< 3 months	27 (4.2)
3–12 months	139 (22)
1–2 years	97 (15)
> 2 years	226 (35)
Duration of arm pain, n (%)	
No arm pain	101 (16)
< 3 months	28 (4.4)
3–12 months	182 (28)
1–2 years	137 (21)
> 2 years	183 (29)
Inpatient stay (days)	4 (3–5)
Number of operated vertebrae	3 (2–4)
Diagnosis, n (%)	
Cervical disc herniation	1 (0.2)
Cervical spinal stenosis	641 (99.8)
Type of surgery, n (%)	
Anterior surgery	294 (46)
Laminectomy without fixation	157 (24)
Laminectomy with fixation	113 (18)
Laminoplasty	14 (2.2)
Combination of laminectomy/laminoplasty and foraminotomy	32 (5.0)
Other/not specified	32 (5.0)

**Table 2:** Baseline and follow-up data for participants who responded to the EMS questionnaires at baseline, 1-, and 2-year follow-up (N = 642). Continuous data are presented as median (IQR) and categorical data are presented as n (%)

Variables	n	Baseline	n	1-year follow-up	P value ^a^	n	2-year follow-up	P value ^b^
EMS	642	14 (12–16)	642	15 (12–17)	< 0.001	642	15 (12–17)	0.8
Severe (5–8)		28 (4.3)		14 (2.2)			16 (2.5)	
Moderate (9–12)		198 (31)		158 (25)			169 (26)	
Mild (13–16)		334 (52)		291 (45)			277 (43)	
Normal (16–18)		82 (13)		179 (28)			180 (28)	
EQ-VAS	605	50 (30–60)	625	60 (42–77)	< 0.001	628	60 (40–75)	0.4
NDI	642	38 (24–50)	640	25 (12–42)	< 0.001	642	26 (12–42)	1.0
NRS neck	601	5 (1–7)	629	2 (0–5)	< 0.001	590	2 (1–5)	0.04
NRS arm	598	5 (2–7)	618	2 (0–5)	< 0.001	586	2 (0–5)	0.4
Use of analgesics	634		632		0.01	629		1.0
Yes		417 (65)		380 (59)			378 (59)	
No		217 (34)		252 (39)			251 (39)	
Walking distance	620		625		< 0.001	621		0.2
< 500 m		308 (48)		245 (38)			260 (40)	
≥ 500 m		312 (49)		380 (59)			361 (56)	
Satisfaction rate			630			624		0.4
Satisfied				360 (56)			355 (55)	
Uncertain				188 (29)			171 (27)	
Dissatisfied				82 (13)			98 (15)	

n = number of individuals with data for each variable.

a Baseline vs 1-year follow-up

b 1-year vs 2-year follow-up

**Table 3:** Baseline and follow-up data for participants who responded to EMS at baseline, 1- , 2-, and 5-year follow-up (N = 347). Continuous data are presented as median (IQR) and categorical data are presented as n (%)

Variables	n	Baseline	n	1-year follow-up	P value ^a^	n	2-year follow-up	P value ^b^	n	5-year follow-up	P value ^c^
EMS	347	14 (12–16)	347	15 (13–17)	< 0.001	347	15 (13–17)	0.9	347	15 (12–17)	0.05
Severe (5–8)		14 (4.0)		4 (1.2)			8 (2.3)			8 (2.3)	
Moderate (9–12)		90 (26)		60 (17)			61 (18)			85 (24)	
Mild (13–16)		190 (55)		164 (47)			159 (46)			148 (43)	
Normal (16–18)		53 (15)		119 (34)			119 (34)			106 (30)	
EQ-VAS	333	50 (30–65)	338	70 (49–80)	< 0.001	342	65 (49–80)	0.8	342	65 (40–80)	0.6
NDI	347	36 (21–49)	347	22 (10–38)	< 0.001	347	22 (8–38)	1.0	347	24 (8–40)	0.5
NRS neck	331	5 (1–7)	343	1 (0–4)	< 0.001	316	2 (1–5)	0.05	316	2 (0–5)	0.02
NRS arm	326	5 (2–7)	337	2 (0–5)	< 0.001	313	2 (0–5)	0.2	313	2 (0–5)	0.04
Use of analgesics	345		339		0.003	344		0.8	347		0.3
Yes		225 (65)		191 (55)			192 (55)			185 (53)	
No		120 (35)		148 (43)			152 (44)			157 (45)	
Walking distance	339		339		< 0.001	339		0.5	338		0.3
< 500 m		158 (46)		118 (34)			113 (33)			128 (37)	
≥ 500 m		181 (52)		221 (64)			226 (65)			210 (60)	
Satisfaction rate								0.8			1.0
Satisfied				213 (61)			208 (60)			211 (61)	
Uncertain				94 (27)			91 (26)			94 (27)	
Dissatisfied				35 (10)			40 (12)			35 (10)	

n = number of individuals with data for each variable.

a Baseline vs 1-year follow-up

b 1-year vs 2-year follow-up

c 2-year vs 5-year follow-up

Correlations between baseline versus 1-year EMS, and baseline versus 2-year EMS were 0.71 (CI 0.67–0.75) and 0.69 (CI 0.64–0.73). Similar findings were seen for the other PROMs (data not shown). There was a statistically significant reduction in the proportion of individuals on sick leave from preoperative to 1 year (P <0.001), but not between 1 and 2 years (P = 0.7) (data not shown).

**Figure 2:**
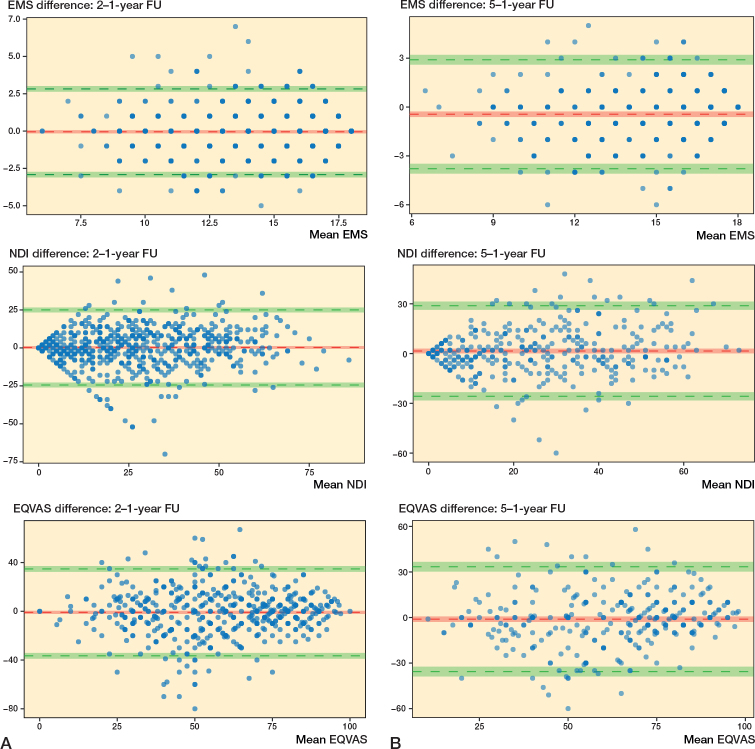
Bland–Altman plots comparing data for EMS, NDI, and EQ-VAS at different times. The y-axis represents the difference between scores, the x-axis represents the mean of both scores. The red horizontal line represents the mean difference and the upper and lower green lines represent the 95% limits of agreement. The 95% confidence intervals are also depicted. (A) 2-year follow-up data compared with 1-year follow-up data (n = 642). (B) 5-year follow-up data compared with 1-year follow-up data (n = 347).

**Figure 3:**
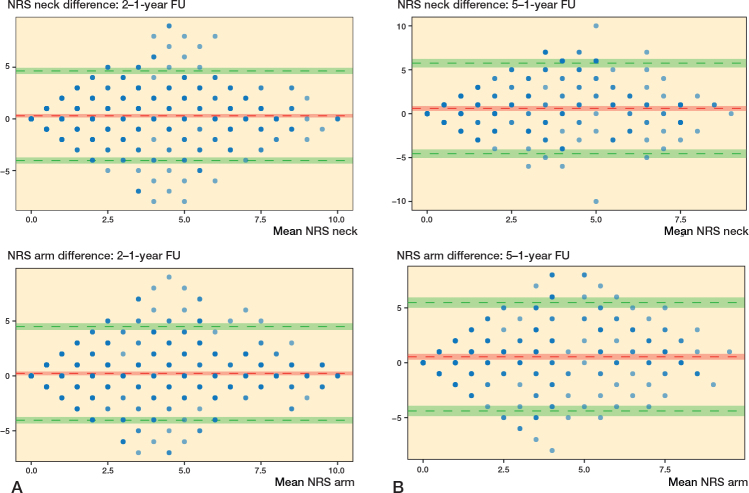
Bland–Altman plots comparing data for NRS. The y-axis represents the difference between scores, the x-axis represents the mean of both scores. The red horizontal line represents the mean difference and the upper and lower green lines represent the 95% limits of agreement. The 95% confidence intervals are also depicted. (A) 2-year follow-up data compared with 1-year follow-up data (n = 642). (B) 5-year follow-up data compared with 1-year follow-up data (n = 347).

In the Bland–Altman plots all 4 PROMs displayed a normal distribution of differences. The 1-year and 2-year data, and 1- and 5-year data for EMS, NDI, EQ-VAS, and NRS indicated good agreement and were without proportional bias (Figure 2 and Figure 3, see Appendix). The differential bias (mean difference) was close to zero for all PROMs, meaning there was no change in PROMs on group level. No statistically significant interaction between the different follow-up time points and the PROMs was found in the mixed ANOVA (data not shown).

Non-responders tended to report poorer results at later follow-ups (Table 4). Of the non-responders at the 2-year follow-up, 40 (15%) had been diagnosed with cervical disc herniation, as opposed to 1 of the responders (0.2%).

**Table 4:** Analysis of non-responder compared with responder data. Continuous data are presented as median (IQR) and categorical data are presented as n (%)

Variables	n	1-year follow-up ^a^ of 2-year responders n = 642	n	1-year follow-up ^b^ of 2-year non-responders n = 259	P value ^c^	n	2-year follow-up ^d^ of 5-year responders n = 347	n	2-year follow-up ^e^ of 5-year non-responders n = 295	P value ^f^
EMS	642	15 (12–17)	259	14 (12–17)	0.2	347	15 (13–17)	295	14 (12–16)	< 0.001
EQ-VAS	625	60 (42–77)	254	60 (38–72)	0.02	339	70 (49–80)	287	57 (40–75)	< 0.001
NDI	640	25 (12–42)	259	28 (12–44)	0.3	347	22 (10–38)	293	28 (16–42)	< 0.001
NRS neck	629	2 (0–5)	251	2 (0–5.5)	0.5	343	1 (0–4)	286	2 (1–6)	< 0.001
NRS arm	618	2 (0–5)	246	3 (0–6)	0.08	337	2 (0–4)	281	3 (1–6)	< 0.001
Use of analgesics	632		256		1.0	339		293		0.04
Yes		380 (59)		154 (60)			191 (56)		189 (65)	
No		252 (39)		102 (40)			148 (44)		104 (35 )	
Walking distance	625		254		0.2	339		286		< 0.001
<500 m		245 (38)		112 (44)			118 (35)		159 (56)	
≥500 m		380 (59)		142 (56)			221 (65)		127 (44)	
Satisfaction rate	630		256		0.7	342		288		0.01
Satisfied		360 (56)		139 (54)			213 (61)		147 (51)	
Uncertain		188 (29)		79 (31)			94 (27)		94 (33)	
Dissatisfied		82 (13)		38 (15)			35 (10)		47 (16)	

n = number of individuals with data for each variable

a Patients who had responded to EMS questionnaires at baseline, 1-year, and 2-year follow-up.

b Patients who had responded to EMS questionnaires at baseline and at the 1-year follow-up, but not at the 2-year follow-up.

c 1-year follow-up: 2-year responders vs 2-year non-responders.

d Patients who had responded to EMS questionnaires at baseline, 1-year, 2-year, and 5-year follow-up.

e Patients who had responded to EMS questionnaires at baseline, 1-year, 2-year, but not at the 5-year follow-up.

f 2-year follow-up: 5-year responders vs 5-year non-responders.

## Discussion

The purpose of our study was to investigate whether there is further improvement in PROMs after the 1-year follow-up after surgery for DCM with data from Swespine. We found that in the entire cohort there were no clinically meaningful changes in PROMs at the 2-year follow-up compared with the 1-year follow-up. All PROMs improved between baseline and the 1-year follow-up, with similar correlations between baseline and the 1- and 2-year follow-up time points, and there was very little or no change in either direction between the 1-year and the 2-year follow-ups.

Regarding EMS, there were some differences regarding proportion of disease severity based on EMS; however, there was virtually no change at all in median EMS score. Regarding these classifications of disease severity, a patient could easily move between these groups based on 1 single point, more or less. For this reason, the classifications do not necessarily reflect the changes as well as the total EMS score, which remained constant over the years.

The PROMs were similar at all follow-up time points, as indicated in both Tables 2 and 3, in Figure 2, Figure 3 in Appendix, in the mixed ANOVA analyses, and the Bland–Altman plots. The limits of agreements for each PROM are, however, higher and lower than their respective MCIDs, thus the variation may be considered to be moderately large, but not so large as to affect the mean.

The patient acceptable symptom state (PASS) is another way of describing the outcome of treatment. However, for the PROMs used, PASSs are not available but satisfaction rate could be seen as a fair substitute and showed little or no variation over time.

The findings of this study are in line with previous studies, where improvements have occurred at the 1-year follow-up, and in some studies already at the 3-month follow-up [2-6]. As the main object of surgical treatment is to halt the progress of myelopathy and decrease the risk of severe neurological symptoms, perhaps further improvement beyond the 1-year follow-up time point should not be expected at all. Even the improvements in PROMs at the 1-year follow-up could be termed an extra advantage that cannot be guaranteed. Although all studies until now seem to find that no relevant change occurs beyond the first year, additional studies on degenerative cervical myelopathy would be welcome.

### Limitations

First, the study is retrospective in nature, and thus it is prone to selection bias. Despite the large number of patients lost to follow-up, the number of included patients is still large. There were a few values missing for the PROMs. Missing data were handled through pairwise exclusion. Other methods to handle missing data exist but are unlikely to change the results.

Furthermore, a significant number of participants were lost to follow-up. Because the results from the 2-year and 5-year-follow-up are based on increasingly smaller cohorts, the results may not be generalizable. Thus, it is possible that these results are not applicable to cohorts with better response rates. However, a study on the importance of loss to follow-up in evaluation after lumbar spine surgery [23] suggests that non-responders achieve similar results to responders. Another limitation is that this study was made within the framework of the Swespine registry, which means variables not included or those insufficiently answered, cannot be used.

### Conclusion

In individuals treated for degenerative cervical myelopathy no clinically meaningful change in PROMs occurred after the 1-year follow-up. Thus, the perspective is that it may not be necessary to obtain PROM follow-up data after the 1-year follow-up.

In the long term it is important to identify additional spine surgeries on these patients. Data on additional surgeries and the reason for them is collected by the surgeon in charge and is independent of collection of PROM data, and not limited in time from the index procedure. Data catchment on additional surgeries can further be improved by using other public registers in Sweden.

## Notes

LG: study design, data management, analysis, first draft of manuscript. PG: study design, supervision, data management, analysis, manuscript revision. AM: study design, supervision, manuscript revision.

The authors would like to acknowledge all the patients and surgeons contributing data to the Swedish Spine Registry, and Carina Blom for help with data extraction.

Handling co-editors: Ilkka Helenius and Philippe Wagner

*Acta* thanks Filip C Dolatowski and Antti Malmivaara for help with peer review of this manuscript.
